# Overall survival of transplant eligible patients with newly diagnosed multiple myeloma: comparative effectiveness analysis of modern induction regimens on outcome

**DOI:** 10.1038/s41408-018-0163-7

**Published:** 2018-12-11

**Authors:** Ashley R. Paquin, Shaji K. Kumar, Francis K. Buadi, Morie A. Gertz, Martha Q. Lacy, Angela Dispenzieri, David Dingli, Lisa Hwa, Amie Fonder, Miriam Hobbs, Suzanne R. Hayman, Steven R. Zeldenrust, John A. Lust, Stephen J. Russell, Nelson Leung, Prashant Kapoor, Ronald S. Go, Yi Lin, Wilson I. Gonsalves, Taxiarchis Kourelis, Rahma Warsame, Robert A. Kyle, S. Vincent Rajkumar

**Affiliations:** 1 Mayo Clinic School of Medicine, Rochester, MN USA; 2 Division of Hematology, Mayo Clinic, Rochester, MN USA

## Abstract

Overall survival (OS) of multiple myeloma has improved remarkably over time, with the recent Intergroupe Francophone du Myelome (IFM) 2009 randomized trial reporting a 4-year OS rate of approximately 82% in patients receiving modern therapy. However, survival estimates from clinical trials may overestimate outcomes seen in clinical practice even with the adjustment for age and other key characteristics. The purpose of this study was to determine the OS of myeloma patients seen in routine clinical practice who resembled the cohort studied in the IFM 2009 trial. A second goal was to conduct a brief comparative effectiveness analysis of bortezomib, lenalidomide, dexamethasone, and other major induction regimens used during the study period. We studied all patients with myeloma 65 years of age and younger, seen at the Mayo Clinic between January 1, 2010 and August 31, 2015, who had a stem cell harvest performed within 12 months of initial diagnosis. Patients with baseline serum creatinine >2 mg/dL were excluded. Five hundred and eighteen patients were studied. The 4-year OS rate was 82.3%, comparable to results achieved in the contemporaneous IFM randomized trial. The 4-year OS rates for standard and high-risk myeloma were 86.3% and 68.2%, respectively.

## Introduction

Multiple myeloma (MM) is a hematologic malignancy of monoclonal plasma cell proliferation, which accounts for 10% of hematologic malignancies and has an annual incidence of approximately 4/100,000^1^. The disease is typically associated with osteolytic bone disease^2^, and is consistently preceded by precursor states of monoclonal gammopathy of uncertain significance (MGUS) and/or smoldering multiple myeloma (SMM)^3,4^. Diagnosis of MM requires 10% or more clonal plasma cells in the bone marrow and/or a biopsy-proven plasmacytoma plus one or more of the following myeloma defining events: evidence of end-organ damage (hypercalcemia, renal insufficiency, anemia, or bone lesions) attributable to the underlying plasma cell disorder, 60% or more clonal bone marrow plasma cells, serum involved/uninvolved free light chain (FLC) ratio ≥100 (provided involved FLC level is ≥100 mg/L), or >1 focal lesion (5 mm or more in size) on magnetic resonance imaging^1^. Prognosis is related to the stage at diagnosis, comorbidities that may impact treatment options, performance status, serum albumin, age, and additional factors^5^. Importantly, cytogenetic abnormalities such as t(4;14), t(14;16), t(14;20), gain 1q, del17p, and p53 mutations are associated with an adverse effect on overall survival (OS), and can be used to stratify patients into low- or high-risk disease^6–9^.

Advances in the treatment of MM have greatly improved OS over the past decade^10^. Standard chemotherapy regimens for initial therapy include combinations such as bortezomib, lenalidomide, dexamethasone (VRd); bortezomib, cyclophosphamide, dexamethasone (VCd); and lenalidomide and dexamethasone (Rd)^6^. New oral regimens for initial therapy are also under investigation^11^. Modern treatment is stratified by eligibility for autologous stem cell transplant (ASCT). In patients that are eligible for ASCT, treatment usually involves 2–4 months of induction chemotherapy followed by ASCT. After transplant, maintenance therapy with lenalidomide or bortezomib is recommended in most patients^6^.

The best OS estimates using modern therapy in transplant eligible patients are derived from the recent Intergroupe Francophone du Myelome (IFM) 2009 trial^12^. This trial showed excellent outcomes, with OS rates in excess of 80% at 4 years. However, data from clinical trials may overestimate the benefit of therapy since patients with poor performance status and serious comorbidities are systematically excluded. Further, these trials are performed under ideal conditions with strict inclusion and exclusion criteria and specific treatment protocols. In contrast, modern medical practice is often complicated by delays in treatment, missed doses, and other confounding factors. Therefore, it is not clear whether the excellent survival of transplant eligible myeloma patients reported in recent clinical trials reflects what is achievable in a general clinical practice setting where only a small proportion of patients are enrolled into clinical trials.

The purpose of this study was to determine if OS of myeloma patients seen in a clinical practice setting matched the results of the IFM 2009 trial. The expansive patient database of the Mayo Clinic provides the opportunity to address this question by studying a cohort of consecutive myeloma patients during a defined time period. Further, few randomized trials have been performed to compare the effect of different pre-transplant induction therapies on survival. Therefore a second goal of the study was to conduct a brief comparative effectiveness analysis of VRd (the regimen used in the IFM trial) with other major induction regimens used during the study period at our institution.

## Methods

### Study cohort

We studied the outcome of consecutive newly diagnosed MM patients seen at Mayo Clinic who resembled the cohort studied in the IFM 2009 trial. To accomplish this, we first queried the Mayo Clinic multiple myeloma database to identify all patients with MM diagnosed at 65 years of age and younger who were seen at the Mayo Clinic between January 1, 2010 and August 31, 2015, and had a stem cell harvest performed within 12 months of initial diagnosis.

Electronic medical records were then reviewed for demographic data, physician notes, laboratory data, imaging studies, and pathology reports including bone marrow aspirates and fluorescent in situ hybridization (FISH) characterization. The following factors were abstracted: diagnosis date, first therapy regimen, ASCT date, progression and survivorship status and date (if applicable), progression reason, details of maintenance therapy, and tumor genetic abnormalities. Additional abstracted data included comprehensive laboratory data collected at initial diagnosis including hemoglobin, calcium, creatinine, serum M spike, kappa and lambda FLC levels, and presence or absence of lytic lesions. We excluded patients with baseline serum creatinine >2 mg/dL. Overall, progression was defined by a medical indication for a change in treatment strategy. For patients who did not achieve first remission with the initial drug regimen, progression was defined by the date of a change of therapy; this was typically due to adverse drug effects or disease progression while on therapy. ASCT performed at first remission was considered to be a single treatment strategy, and was not considered to be a progression. Approval for this study was obtained from the Mayo Clinic Institutional Review Board according to federal regulations and in accordance with the Declaration of Helsinki.

### Molecular cytogenetic classification

All cIg-FISH studies were performed for clinical purposes at the Mayo Clinic, Rochester, MN, USA as previously described^13–15^. Briefly, aspirate samples were enriched for mononuclear cells using ACK lyse, and cytospin slides were prepared. FISH analysis was performed using the following probes: 3cen (D3Z1), 7cen (D7Z1), 9cen (D9Z1), 15cen (D15Z4), 11q13 (CCND1-XT), 14q32 (IGH-XT), 13q14 (RB1), 13q34 (LAMP1), 14q32 (5′IGH,3′IGH), 17p13.1 (p53), and 17cen (D17Z1). Additional probes as needed were used to detect t(4;14), t(14;16), t(14;20), and other abnormalities based on the results of the initial screen. For the purposes of this study, the presence of trisomies of one or more odd-numbered chromosomes was classified as trisomies. A patient was classified into the specific trisomies and immunoglobulin heavy chain (IgH) translocation categories regardless of when these abnormalities were detected in the course of the disease as previously described, including after progression to MM, since these abnormalities are considered primary and present from the initial MGUS stage^13,16^. Conversely, monosomy 13/del(13q) and del(17p) were considered only if they were detected within 1 year of diagnosis. Patients were initially classified into non-overlapping primary cytogenetic groups: trisomies, t(11;14), t(4;14), t(6;14), MAF translocations [t(14;16) or t(14;20)], unknown/other IgH translocation partner, both trisomies and IgH translocations, monosomy 14 in the absence of any other primary cytogenetic abnormality, and normal or insufficient plasma cells^6^. Subsequently, groups were pooled together for additional analyses. The presence of del 17p, (14;16), (14;20), and (4;14) was defined as high-risk cytogenetics. If a high-risk cytogenetic abnormality was present, it was considered to be dominant and the patient was categorized overall as having high risk. All other patients were considered to have standard-risk cytogenetics.

### Statistical analysis

Two-sided Fisher exact tests were used to test for differences between categorical variables. Two-sided Wilcoxon rank-sum tests were used to compare continuous variables. Kaplan–Meier analysis was performed to generate survival curves. Groups were compared with the two-tailed log-rank test. Multivariate analysis was performed using Cox’s proportional hazards model. Median follow up time was calculated using the reverse Kaplan–Meier method.

## Results

### Patient characteristics

A total of 518 patients (285 men and 233 women) were studied. Patient characteristics at diagnosis are shown in Table 1. The median follow-up duration was 50.8 months (range, 5.1–90.1). Median age at diagnosis was 57.9 years (range, 32.8–65.0).

**Table 1:** Patient characteristics

Characteristic	Number of patients (%) (*N*=)
Male gender	285 (55.0)
Progression of disease	337 (67.0)
Deaths	110 (21.2)
**First regimen**	
Lenalidomide, dexamethasone (Rd)	150 (29.0)
Bortezomib, cyclophosphamide, dexamethasone (VCd), *n* (%)	144 (27.8)
Bortezomib, lenalidomide, dexamethasone (VRd)	134 (25.9)
Bortezomib, dexamethasone (Vd)	42 (8.1)
Ixazomib, cyclophosphamide, dexamethasone (ICd)	16 (3.1)
Carfilzomib, lenalidomide, dexamethasone (KRd)	8 (1.5)
Ixazomib, lenalidomide, dexamethasone (IRd)	3 (0.6)
Other	21 (4.1)
**Cytogenetics (data available in 482 patients)**	
High risk	113 (23.4)
Standard risk	369 (76.6)

### Cytogenetic abnormalities

Cytogenetic data was available for 482 patients; 113 patients (23.4%) had high-risk disease and 369 patients (76.6%) had standard-risk disease. The detailed distribution of cytogenetic abnormalities is shown in Table 2. Overall, 153 patients (31.7%) had IgH translocations, 175 (36.3%) had trisomies, and 77 (15.9%) had both trisomies and IgH translocations. Eight patients (1.7%) had monosomy 14 in the absence of other abnormalities. There were no patients with del 17, monosomy 13, or del 13q without other concurrent primary abnormalities.

**Table 2:** Distribution of cytogenetic abnormalities

Cytogenetic abnormality	Number of patients (%)^a^
Primary cytogenetic abnormalities	
t(11;14)	90 (18.7)
t(4;14)	50 (10.4)
t(6;14)	5 (1)
t(14;16)	19 (3.9)
t(14;20)	7 (1.5)
t(14;other)	54 (11.2)
Trisomies	254 (52.7)
Monosomy 14	8 (1.7)
Secondary cytogenetic abnormalities	
Del 17p	49 (10.2)
Monosomy 13	185 (38.4)
Del 13q	16 (3.3)
Other	22 (4.6)
Insufficient cells or no abnormalities detected	45 (9.3)
**Total**	**482**

^a^Total does not equal 482 or 100% due to overlap in cytogenetic abnormalities

### Therapy

Details of Initial therapy are provided in Table 3. The most common regimens used were Rd (*n* = 150), VCd (*n* = 144), and VRd (*n* = 134). Overall, 192 patients (37.1%) received doublet therapy, with Rd or bortezomib plus dexamethasone (Vd). Three hundred and five patients (58.9%) received triplet therapy. Of these, an immunomodulatory agent–proteasome inhibitor containing triplet was used in 145 patients (28%) and a proteasome inhibitor–alkylating agent containing triplet was used in 160 patients (30.9%). Comparative effectiveness analysis was performed among the 3 major groups: patients receiving doublet therapy, immunomodulatory agent–proteasome inhibitor containing triplet therapy, and proteasome inhibitor–alkylating agent containing triplet therapy. Only 21 patients (4.1%) received other regimens.

**Table 3:** Breakdown of induction regimen

Regimen	Number of patients (%)
**IMID/PI**	145 (30%)
VRd	134 (25.9%)
KRd	8 (1.5%)
IRd	3 (.5%)
**PI/alkylator**	160 (30.9%)
VCd	144 (27.8%)
ICd	16 (3.1%)
**Doublet**	192 (37.1%)
Rd	150 (29%)
Vd	42 (8.1%)
**Other**	21 (4.1%)

Abbreviations: *VRd* bortezomib, lenalidomide, dexamethasone; *KRd* carfilzomib, lenalidomide, dexamethasone; *IRd* ixazomib, lenalidomide, dexamethasone; *PI* proteasome inhibitor; *VCd* bortezomib, cyclophosphamide, dexamethasone; *ICd* ixazomib, cyclophosphamide, dexamethasone; *Rd* lenalidomide, dexamethasone; *Vd* bortezomib, dexamethasone

### Maintenance

For the 390 patients for whom we had enough follow up information to determine if maintenance was used, 216 (55%) received maintenance. One hundred and thirty-one patients received lenalidomide with or without dexamethasone, 78 received bortezomib with or without dexamethasone, 3 patients received thalidomide, 2 patients received ixazomib, 1 patient received carfilzomib, and 1 received bortezomib and lenalidomide.

### Survival

**Fig. 1:**
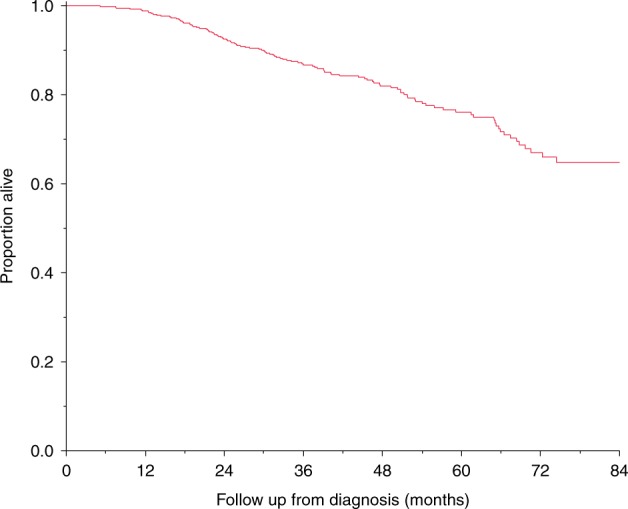
Overall survival. The 4-year overall survival rate of the whole cohort was 82%; 5-year overall survival rate was 76.1%

**Fig. 2:**
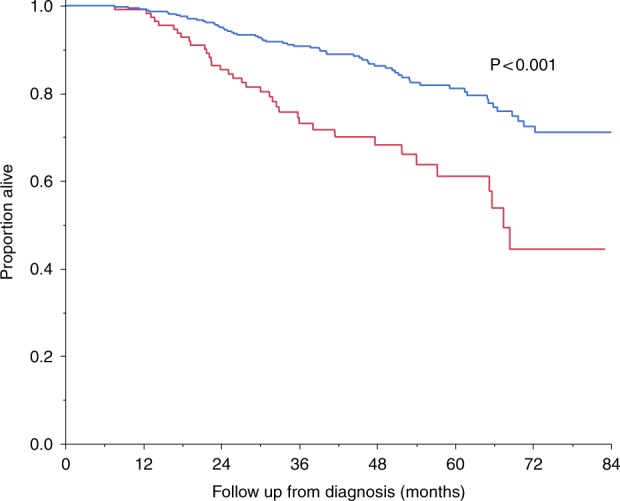
Overall survival by cytogenetic risk stratification. Median overall survival not reached (standard-risk myeloma; blue curve) versus 67.4 months (high-risk myeloma; red curve), *P* < 0.001; corresponding 4-year overall survival rates were 86.3% and 68.2%, respectively

The median OS has not been reached (Fig. 1). The 4-year OS rate of the whole cohort was 82.3%; 5-year OS rate was 76.1%. Survival was significantly superior for standard-risk myeloma versus high-risk myeloma patients, median OS not reached versus 67.4 months, respectively, *P* < 0.001 (Fig. 2). Corresponding 4-year OS rates were 86.3% and 68.2%, respectively.

**Fig. 3:**
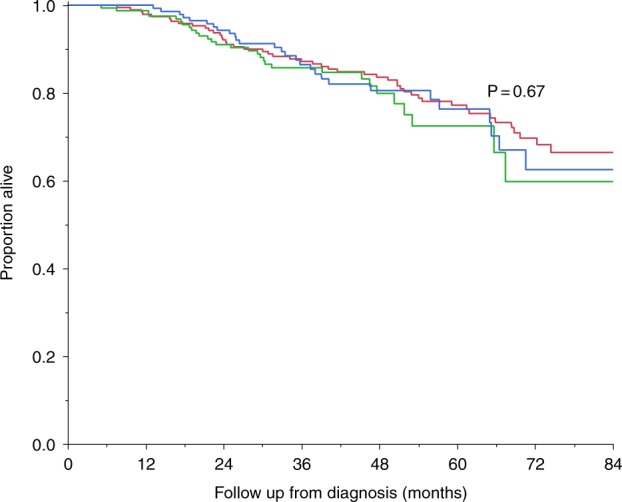
Overall survival by induction treatment regimen. 4-year overall survival rates with immunomodulatory drug–proteasome inhibitor combination (*n* = 145; blue curve), proteasome inhibitor–alkylator combination (*n* = 160; green curve), and a doublet regimen (*n* = 192; red curve) were similar, 80.6%, 79.9%, and 83.6%, respectively, *P* = 0.67

The 4-year OS rates for patients receiving an immunomodulatory drug–proteasome inhibitor combination (*n* = 145), proteasome inhibitor–alkylator combination (*n* = 160), and a doublet regimen (*n* = 192) were similar, 80.6%, 79.9%, and 83.6%, respectively, *P* = 0.67 (Fig. 3). High-risk myeloma patients were, however, more likely to be treated with a triplet combination, consistent with our clinical practice during the period of the study. Thus, among patients with high-risk MM (*n* = 110), 48 received an immunomodulatory drug–proteasome inhibitor combination, 39 received a proteasome inhibitor–alkylator combination, and only 23 received doublet therapy. In contrast, among patients with standard-risk MM (*n* = 352), 86 received an immunomodulatory drug–proteasome inhibitor combination, 113 received proteasome inhibitor–alkylator combination, and 153 received doublet therapy. The 4-year OS rates with these regimens stratified by cytogenetic risk classification are shown in Table 4. Adjusting for cytogenetic risk, the OS between the 3 regimen classes remained non-significant, *P* = 0.93.

**Table 4:** Four-year survival rates of patients with newly diagnosed myeloma based on initial treatment and myeloma risk classification

Risk group	IMiD/PI (*n* = 134)	PI/Alkylator (*n* = 152)	Doublet (*n* = 176)
Standard-risk myeloma (*n* = 352)^a^	84.7%	83.4%	87.6%
High-risk myeloma (*n* = 110)^a^	70.2%	73.0%	60.9%

*IMiD/PI* immunomodulatory drug–proteasome inhibitor containing triplet combination; *PI/Alkylator* proteasome inhibitor–alkylating agent containing triplet combination

^a^Among patients with high-risk myeloma (*n* = 110), 48 received IMiD/PI, 39 PI/Alkylator, and 23 doublet. Among patients with standard-risk myeloma (*n* = 369), 86 received IMiD/PI, 113 PI/Alkylator, and 153 doublet

**Fig. 4:**
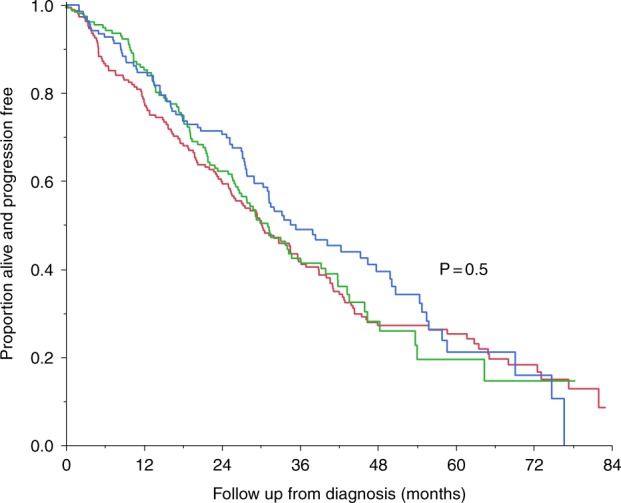
Progression-free survival by induction treatment regimen. Progression-free survival with immunomodulatory drug–proteasome inhibitor combination (*n* = 145), proteasome inhibitor–alkylator combination (*n* = 160), and a doublet regimen (*n* = 192) were similar. Median progression-free survival was 35.4 months (immunomodulatory drug–proteasome inhibitor combination; blue curve), 31.2 months (proteasome inhibitor–alkylator combination; green curve), and 30.1 months (doublet regimen; red curve), *P* = 0.5

Progression-free survival (PFS) was not significantly different between the three groups, with median PFS 30.1 months (doublet), 31.2 months (proteasome inhibitor–alkylator combination), and 35.4 months (immunomodulatory drug–proteasome inhibitor combination), *P* = 0.5 (Fig. 4).

## Timing of transplantation

All patients per inclusion criteria were transplant eligible, and had stem cell collected within 12 months of diagnosis. Of these, 282 patients (54.4%) underwent early ASCT. Of those opting for a delayed ASCT (*n* = 236), 56 patients have undergone transplantation at the time of this analysis. On intent to treat analysis, the 4-year OS rate was 82.1% among patients undergoing early ASCT versus 81.8% among patients intending for delayed ASCT, *P* = 0.75. The median PFS was 32.7 months versus 31 months, respectively, *P* = 0.86.

## Discussion

We found that the survival of transplant eligible patients under age 65 treated at Mayo Clinic was similar to the exceptional results observed in a recent prospective randomized clinical trial conducted by the IFM in a similar patient population^12^. The 4-year OS in our study was 82%, identical to that reported in the IFM trial. Results from randomized trials may overstate the benefit with treatment since they are based on studies on highly selected patients who meet stringent eligibility criteria, and are therefore may not generalizable to the general population. Our results show that the gains in myeloma survival are indeed generalizable, demonstrating similar survival rates with minimal selection criteria. We chose consecutive patients seen at Mayo Clinic who met minimal thresholds for rough comparability to the IFM trial (age, transplant eligibility, and creatinine <2.0 mg/dL) but did not use any other criteria to select or exclude patients. These results represent a continued improvement over the results reported in earlier studies by our group and others^10,17,18^. As with the IFM trial, we found no differences in patients who opted for early ASCT versus those who decided to delay the procedure, 4-year survival rate 82.1% versus 81.8% respectively, *P* = 0.75. However, unlike the IFM trial, we did not find significant differences in PFS between the groups, but this may reflect a selection bias because assignment to the groups was not random, and patients not responding adequately to initial therapy may have been more likely to be offered early ASCT.

As expected, survival in myeloma continues to be affected by underlying cytogenetic risk stratification (Fig. 2). Of note, the 4-year OS rate was 68.2% in patients with high-risk disease. A recent update of the HOVON trial found that there may be heterogeneity in the outcome among patients classified as high-risk myeloma. In that trial, the 8-year OS rate was 30% among patients with t(4;14) and gain(1q), but 50% among patients with del(17p)^19^. Our sample size is small to analyze the outcome by cytogenetic type, and we will continue to study this in future cohorts. Most patients in our cohort did not also benefit from the systematic used of bortezomib-based maintenance as was done in the HOVON trial; only 79 patients could be confirmed to have used a bortezomib-based maintenance regimen. This represents 20% of patients for whom maintenance could be confirmed or denied, and 15% of all patients. Further, the results of our study do not include more recent gains in myeloma therapy, especially the introduction of daratumumab and other immunotherapeutic approaches^20,21^. We are hopeful that with these additions the outcome of high-risk myeloma will improve and approach that seen in standard-risk MM. However, additional new treatments for myeloma are needed since our results show no plateau in the PFS curves, confirming other observations by our group on the lack of curability in myeloma with current treatments^22^.

We found no significant difference in PFS or OS by the type of initial therapy regimen used. However, since we have used a risk-adapted approach to therapy at Mayo Clinic^23^, we cannot directly compare the regimens since high-risk myeloma patients were more likely to be treated with a triplet combination, while more patients with standard-risk myeloma received doublet therapy. It is possible that for transplant eligible patients with good availability of salvage regimens, OS is not affected by choice of induction therapy. Thus after adjusting for cytogenetic risk, the OS between the 3 regimen classes remained non-significant, *P* = 0.93. However, the added toxicity of VRd compared with a doublet regimen is minimal, and we defer to the results of the Southwest Oncology Group trial^24^, and continue to recommend VRd as the standard regimen for initial therapy for most patients with newly diagnosed myeloma.

One limitation of our study is that it comes from a major referral center, and hence although we did not employ rigorous inclusion and exclusion criteria, patients studied may represent a selected subset not representative of general practice in patients of similar age in the US. We also included only patients who were considered “transplant eligible” and this may have excluded younger patients with poor performance status. Additionally, since a primary goal of this study was to compare the survival of our patients to those in the IFM trial, we also excluded patients with a creatinine greater than 2, which excluded those patients with poor renal status. More data are needed to examine these effects in specific cytogenetic groups. Finally, follow up is still short, and we will continue to observe this cohort to determine whether significant survival differences emerge with increased follow up between groups such as early versus delayed transplant.

We conclude that transplant eligible patients with MM have a 4-year survival probability in excess of 80%. Our findings will have an impact in accurate counseling of patients.

## References

[1] Rajkumar SV (2014). International Myeloma Working Group updated criteria for the diagnosis of multiple myeloma. Lancet Oncol..

[2] Terpos E, Ntanasis-Stathopoulos I, Gavriatopoulou M, Dimopoulos MA (2018). Pathogenesis of bone disease in multiple myeloma: from bench to bedside. Blood Cancer J..

[3] Kyle RA (2018). Long-term follow-up of monoclonal gammopathy of undetermined significance. N. Engl. J. Med..

[4] Kyle RA (2007). Clinical course and prognosis of smoldering (asymptomatic) multiple myeloma. N. Engl. J. Med..

[5] Palumbo A (2015). Revised international staging system for multiple myeloma: a report from International Myeloma Working Group. J. Clin. Oncol..

[6] Kumar SK, Rajkumar SV (2018). The multiple myelomas—current concepts in cytogenetic classification and therapy. Nat. Rev. Clin. Oncol..

[7] Binder M (2017). Prognostic implications of abnormalities of chromosome 13 and the presence of multiple cytogenetic high-risk abnormalities in newly diagnosed multiple myeloma. Blood Cancer J..

[8] Chavan SS (2017). Bi-allelic inactivation is more prevalent at relapse in multiple myeloma, identifying RB1 as an independent prognostic marker. Blood Cancer J..

[9] Chin M (2017). Prevalence and timing of TP53 mutations in del(17p) myeloma and effect on survival. Blood Cancer J..

[10] Kumar SK (2014). Continued improvement in survival in multiple myeloma: changes in early mortality and outcomes in older patients. Leukemia.

[11] Kumar SK (2018). Phase 1/2 trial of ixazomib, cyclophosphamide and dexamethasone in patients with previously untreated symptomatic multiple myeloma. Blood Cancer J..

[12] Attal M (2017). Lenalidomide, bortezomib, and dexamethasone with transplantation for myeloma. N. Engl. J. Med..

[13] Rajkumar SV (2013). Impact of primary molecular cytogenetic abnormalities and risk of progression in smoldering multiple myeloma. Leukemia.

[14] Kumar S (2012). Trisomies in multiple myeloma: impact on survival in patients with high-risk cytogenetics. Blood.

[15] Fonseca R (2002). Biological and prognostic significance of interphase fluorescence is situ hybridization detection of chromosome 13 abnormalities in multiple myeloma: an Eastern Cooperative Oncology Group Study. Cancer Res..

[16] Greenberg AJ (2014). Relationship between initial clinical presentation and the molecular cytogenetic classification of myeloma. Leukemia.

[17] Kumar SK (2008). Improved survival in multiple myeloma and the impact of novel therapies. Blood.

[18] Kastritis E (2009). Improved survival of patients with multiple myeloma after the introduction of novel agents and the applicability of the International Staging System (ISS): an analysis of the Greek Myeloma Study Group (GMSG). Leukemia.

[19] Goldschmidt H (2018). Bortezomib before and after high-dose therapy in myeloma: long-term results from the phase III HOVON-65/GMMG-HD4 trial. Leukemia.

[20] Rajkumar SV, Kyle RA (2016). Progress in myeloma—a monoclonal breakthrough. N. Engl. J. Med..

[21] Rajan AM, Kumar S (2016). New investigational drugs with single-agent activity in multiple myeloma. Blood Cancer J..

[22] Ravi P (2018). Defining cure in multiple myeloma: a comparative study of outcomes of young individuals with myeloma and curable hematologic malignancies. Blood Cancer J..

[23] Mikhael JR (2013). Management of newly diagnosed symptomatic multiple myeloma: updated Mayo Stratification of Myeloma and Risk-Adapted Therapy (mSMART) Consensus Guidelines 2013. Mayo Clin. Proc..

[24] Durie BGM (2017). Bortezomib, lenalidomide and dexamethasone vs. lenalidomide and dexamethasone induction followed by lenalidomide and dexamethasone maintenance in patients with newly diagnosed myeloma without intent for immediate autologous stem cell transplant: results of the Randomised Phase III SWOG Trial S0777. Lancet.

